# Lipemia Interferences in Biochemical Tests, Investigating the Efficacy of Different Removal Methods in comparison with Ultracentrifugation as the Gold Standard

**DOI:** 10.1155/2020/9857636

**Published:** 2020-02-12

**Authors:** Neda Soleimani, Sahand Mohammadzadeh, Fateme Asadian

**Affiliations:** ^1^Department of Pathology, Shiraz University of Medical Sciences, Shiraz, Iran; ^2^Department of Medical Laboratory Sciences, School of Paramedical Sciences, Shiraz University of Medical Sciences, Shiraz, Iran

## Abstract

*Introduction*. As a common interferer in clinical chemistry, lipemic specimens could be a source of significant analytical errors. Ultracentrifugation has been by far the only reliable, but an unavailable and expensive, method to eliminate the lipemic effect. *Materials and Methods*. Among the daily samples, those with triglyceride >400 mg/dL (4.6 mmol/L) and also turbid were selected, divided into three groups, based on triglyceride concentration, and three pooled serums were made for each group. Then all pooled serums were investigated by using a DIRUI biochemistry analyzer CS-800 for routine chemistry tests in different methods including direct measurement, serum blank, serum dilution, and measurement after ultracentrifugation.

**Results:**

According to our study, there were significant differences before and after ultracentrifugation in all lipemic levels and for all parameters except for alanine aminotransferase (ALT), alkaline phosphatase (ALP), bilirubin, and uric acid. Based on allowable inaccuracy for each parameter, calcium, magnesium, phosphorus, total protein, iron, total iron-binding capacity (TIBC), urea, and chloride are being influenced by all lipemic degree and neither serum dilution nor using serum blank is as effective as ultracentrifuge for elimination. Serum blank was a proper method of lipid removal for the measurement of glucose.

**Conclusion:**

Lipemia is a well-known interferer in clinical chemistry. One cannot avoid lipemia, but fortunately, severe lipemia is a rare phenomenon in the laboratory, and for assessment of some analytes in a lower degree of lipemia, use of serum blank eliminates the need for ultracentrifuge.

## 1. Introduction

Published data states that up to 80% of patient care decisions are based on laboratory data [1]. Nowadays, reducing laboratory errors and improving patient safety are receiving a lot of attention [2]. Preanalytical phase encompasses all the procedures before the start of laboratory testing.

This phase of the testing process is responsible for the majority of laboratory errors [3]. A lot of these errors can link to the analytical sample integrity, of which lipemia is a contributor [4].

Lipemia occurs when serum triglyceride (TG) levels exceed 400 mg/dL (4.6 mmol/L) [5]. The overall frequency of lipemic samples ranges from 0.5 to 2.5%, depending on the type of hospital and the proportion of inpatient and outpatient samples [6–8].

The most common cause of lipemia is short fasting time. Other common causes include genetic background, diabetes mellitus, acute pancreatitis, renal failure, alcoholism, hypothyroidism, and some drugs. Lipemia can cause interference in biochemistry results through a variety of mechanisms such as interference in spectrophotometric methods (probably the most common way of interference), heterogeneity of the sample, and volume displacement effect.

Unlike for other interferences, lipemia can be removed, and measurement can be done in a clear sample. There are several ways of removing lipids: centrifugation (ultra and high speed), lipid extraction (using polar solvents), sample dilution, and serum blank [5, 9, 10].

Although ultracentrifugation is the recommended procedure according to the CLSI, most laboratories do not have access to that because of the high cost [11]. That is why it is crucial to determine if there are other more accessible and more practical methods to remove lipemia in routine biochemical tests. Many reagent suppliers provide information on the effect of lipemia in their assays, but this is often vague, is not quantified, and may not be instrument-specific [12]. Neither all analytes nor all levels of lipemia are susceptible to lipemia interference. Accordingly, it seems to be necessary to choose method-dependent and parameter-dependent ways for lipid removal which also should be compatible with the level of lipemia.

Our study is novel in which we evaluated the effect of sample dilution and serum blank compared to ultracentrifugation for lipid removal in different levels of lipemia and 21 biochemical parameters.

## 2. Materials and Methods

### 2.1. Samples

This study was conducted in the clinical laboratory of Shahid Motahari Clinic (an outpatient department of Shiraz University of Medical Sciences) from August to September of 2018. The study was approved by the Ethics Committee of the university. Among more than 1000 daily samples of the laboratory, about 1-2% of them have lipemic serums. To study the effect of lipemia on routine biochemistry tests, we selected the visibly turbid serums with a TG concentration of >400 mg/dL (4.6 mmol/L). A total of 208 serums were collected of which 6 were excluded from the study due to concurrent hemolysis or icterus. Specimens TG concentration ranged from 401 to 3562 mg/dL.

### 2.2. Methods

The specimens were divided into three groups according to TG level in mg/dL (mild lipemia: 400–700, moderate lipemia: 700–1000, and severe lipemia: >1000). Three pooled serums were made for each group and all pooled serums were run for 21 parameters, on the DIRUI biochemistry analyzer CS-800 directly and also with three other different methods, including serum dilution (1/10 with distilled water, automatically), use of serum blank option of autoanalyzer, and direct measurement after ultracentrifugation (at 100,000 ×*g* for 15 min) in the same batch. Biochemistry parameters consisted of alanine aminotransferase (ALT), albumin, alkaline phosphatase (ALP), aspartate aminotransferase (AST), amylase, bilirubin (total), calcium, chloride, creatine phosphokinase (CPK), creatinine, γ-glutamyl transferase (GGT), glucose, iron, lactate dehydrogenase (LDH), lipase, magnesium, phosphorus, total protein, total iron-binding capacity (TIBC), urea, and uric acid.

### 2.3. Statistical Analysis

The mean and standard deviation were calculated for each parameter in all groups of lipemia and also for all four methods. To compare the mean results of each group, Student's *t*-test was used. Finally, as a reference method for lipid removal, the results of all methods were compared with ultracentrifugation in each group. The percentage of differences (bias) was calculated to determine the effectivity of methods in lipid removal compared with allowable inaccuracy (bias) [13]. The results of TG and cholesterol measurements were eliminated from the study since, due to lipid removal, they were unreliable.

## 3. Result

**Figure 1:**
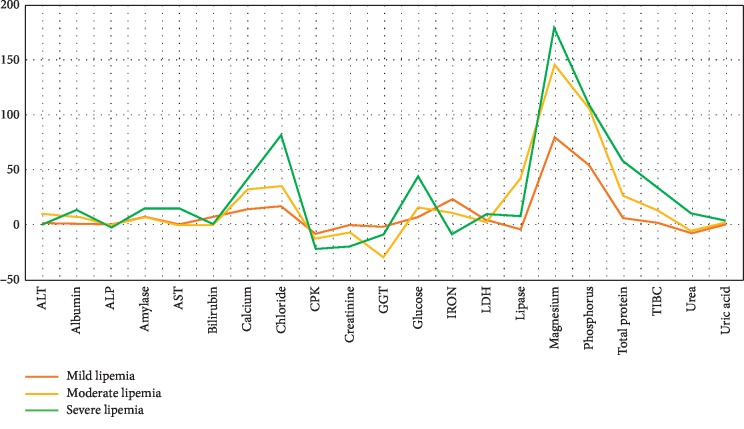
Percentage of bias for 21 biochemistry analytes in different levels of lipemia.

A total of 21 parameters were evaluated in 202 serum samples in three ranges of lipemia (TG: 400–700, TG: 700–1000, and TG: >1000 mg/dl) by spectrophotometric methods (Figure 1). The results of native serum, serum blank, and diluted serum were compared with ultracentrifuge (as a reference method) and the differences were calculated as bias (percentage of difference). For the parameters that the bias between native serum and ultracentrifuged serums does not exceed allowable bias, serum blank and dilution were not applicable (Table 1).

Table 2 shows the bias obtained in mild lipemia (400–700 mg/dl) based on ultracentrifuged samples in comparison with allowable bias. In this range of lipemia, calcium, chloride, glucose, iron, magnesium, phosphorus, total protein, TIBC, and urea had a significant bias in results (*p* value < 0.05 for calcium, phosphorus, and magnesium). In this group, using serum blank was helpful for glucose and chloride measurement, unlike serum dilution.

In moderate lipemia (700–1000 mg/dl), as shown in Table 3, only ALT, ALP, amylase, AST, bilirubin, and uric acid are not influenced by lipemia. Using serum blank was helpful for lipid elimination in the measurement of albumin, CPK, creatinine, glucose, and GGT, but serum dilution was not successful for removal of lipid interference in any of these analytes.

According to Table 4 in severe lipemia (>1000 mg/dl), only ALT, ALP, bilirubin, lipase, and uric acid had not been affected. Using serum blank was practical for lipid elimination in the measurement of albumin, amylase, CPK, and glucose, and serum dilution was useful for removal of lipid interference in creatinine analyte.

## 4. Discussion

In clinical chemistry, pre- and postanalytical factors are the largest and the most important source of errors in comparison with analytical elements. Preanalytic errors are even more common than the postones, so effective correction of interference is recommended to release reliable results. Analytical interference is the effect of substances other than the analyte reacting with the reagents or detection system of the analytical method [14].

The interference by hemolysis, icterus, paraproteinemia, and lipemia is of main concern in the laboratory. Lipemia is a common problem of the specimens. According to the National Cholesterol Education Program Adult Treatment Panel (NCEP ATP III) guidelines, an average TG level is < 150 mg/dl, but just extracted serums with TG > 400 mg/dL lead to visible turbidity and interference [15]. This level of TG could be due to short fasting time, ingestion of fatty meals, drugs (such as cholestyramine, estrogens, and oral contraceptives), alcohol, recent exercise, and pregnancy in addition to genetic predisposition [16].

Lipemia may interfere in any assay which uses the transmission of light as part of the detection scheme and cause increased absorption of light. Lipemia can also cause interferences by volume displacement and heterogeneity of the sample. To evaluate the susceptibility of methods to interferences from icterus or hemolysis, it is appropriate to prepare reference samples with added bilirubin and hemoglobin, respectively, but in the face of lipemia, there is no standardized material and method [17–19].

To prevent the interference of lipemia, the patient should fast for at least 12 to 14 hours before the test, not drink alcohol for 24 hours, or take any fatty diet, discontinuation of any offending medications should be considered as well, and if there was still a lipemic serum, we have to look for a way to eliminate it [20].

Available methods to remove lipids consist of ultracentrifuge (as the gold standard), high-speed centrifuge, lipid extraction (using polar solvents), sample dilution, and serum blank [12].

Ultracentrifuge is an expensive method of lipid removal, which is also unavailable for many laboratories; instead, serum dilution and serum blank are easy and routine methods for removing interferences.

According to previous studies, high-speed centrifuge is almost as effective as ultracentrifuge, but lipid extraction methods do not always work [2, 21]. We investigated which parameters are more susceptible to lipemia of sample and whether the current reference method, ultracentrifugation, could be replaced with a technique that is more available and cheaper to remove lipemia in serum/plasma samples. A large number of parameters were analyzed, and the methods most commonly used for lipemia removal in laboratories were compared.

According to our study, magnesium was responsible for the most significant interference among all analytes and all degrees of lipemia. We found significant differences before and after ultracentrifugation in all lipemic levels and for all parameters except for ALT, ALP, bilirubin, and uric acid.

Calcium, magnesium, phosphorus, total protein, iron, TIBC, urea, and chloride are being influenced by all lipemic degree and neither serum dilution nor using serum blank is effective for elimination, so for measurement of these parameters, just ultracentrifuge is reliable.

Using serum blank is as successful as ultracentrifuge in lipid removal for the measurement of glucose. LDH and AST are just affected by severe lipemia, which is uncorrectable unless using an ultracentrifuge.

In lipase measurement, there is incurable interference in moderate and severe lipemia. About GGT and creatinine, there is an intervention in moderate and severe lipemia and using serum blank helps to be carried away in average levels. About albumin, CPK, and amylase, although mild lipemia has no effects on results, using serum blank is applicable for all lipemic levels.

Till now, some studies have investigated the effect of lipemia on biochemistry parameters with variable results. One study designed by Randall et al. showed lipemia interferences with determination of glucose, phosphorus, total bilirubin, uric acid, and total protein by the Beckman Synchron CX5 [22]. Falsely low levels of amylase and rarely lipase are also seen in lipemic samples [23, 24]. According to Agarwal study, glucose and albumin are not affected by lipemia [25]. Another study by Biljali et al. showed significant differences before and after ultracentrifugation in all analytes except total bilirubin, glucose, total protein, and AST [12]. Although some studies investigated the effect of lipemia on routine biochemical tests, none of them mentioned the importance of the amount of lipemia [26].

These inconsistencies are because the effect of lipemia on biochemical tests is analyte-, method-, and analyzer dependent, and also some autoanalyzers perform an initial blank reading at the start of the reaction [12]. Therefore, every laboratory should determine the amount of lipemia interactions depending on equipment and have a protocol for resolving them.

## 5. Conclusion

One cannot avoid lipemia, but fortunately, severe lipemia is a rare phenomenon in the laboratory, and for assessment of some analytes in a lower degree of lipemia, use of serum blank eliminates the need for ultracentrifuge.

## Figures and Tables

**Table 1:** Chemical methods, wavelength, and analytical range used for analysis.

Parameter	Method	Wavelength (nm)	Analytical range	Reagent
AST	IFCC	340	3–300 IU/L	BIOREX
Bilirubin (total)	Diazo	546	0.1–30 mg/dL	BIOREX
Calcium	CPC	570	0.2–20 mg/dL	BIOREX
Chloride	Colorimetric (thiocyanate)	456	25–300 mmol/L	BIOREX
CPK	IFCC	340	10–1700 IU/L	BIOREX
Creatinine	Jaffe	500	0.2–20 mg/dL	BIOREX
GGT	Szasz IFCC	405	2–231 IU/L	BIOREX
Glucose	Glucose oxidase	546	5–400 mg/dL	BIOREX
Iron	Ferrene	600	5–500 *μ*g/dL	BIOREX
LDH	DGKC	340	50–1200 IU/L	BIOREX
Lipase	Enzymatic	580	0–400 IU/L	BIOREX
Magnesium	Photometric (xylidyl blue)	546	0.5–5 mg/dL	BIOREX
Phosphorus	Ammonium phosphomolybdate	340	0.76–20 mg/dL	BIOREX
TIBC	Ferrene	600	70–700 *μ*g/dL	BIOREX
Total protein	Biuret	546	0.5–15 g/dL	BIOREX
Urea	Urease	340	10–300 mg/dL	BIOREX
Uric acid	Enzymatic	555	0.5–25 mg/dL	BIOREX

**Table 2:** Comparison of the results of native serum, serum blank, and diluted serum with ultracentrifuged serum.

Parameter	Mild lipemia (TG: 400–700 mg/dl)			Allowable bias (%)
	Native serum (%)^*∗*^	Serum blank (%)^*∗*^	Diluted serum (%)^*∗*^	
ALT	+1			**11.48**
Albumin	+1			**1.43**
ALP	0			**6.72**
Amylase	+7			**7.4**
AST	0			**6.54**
Bilirubin (total)	+6			**8.95**
Calcium	+14	+19	+26	**0.82**
Chloride	+17	+2	+21	**0.5**
CPK	−9			**11.5**
Creatinine	0			**3.96**
GGT	−2			**11.6**
Glucose	+7	+1	+26	**2.34**
Iron	+23	+13	+48	**8.8**
LDH	+4			**4.3**
Lipase	−5			**11.31**
Magnesium	+80	+80	+100	**1.8**
Phosphorus	+54	+54	+69	**3.38**
Total protein	+6	−18	+15	**1.36**
TIBC	+2	+3	+24	**1.3**
Urea	−8	−10	−21	**5.57**
Uric acid	0			**4.87**

^*∗*^Difference with ultracentrifuged serum, calculated as bias.

**Table 3:** Comparison of the results of native serum, serum blank, and diluted serum with ultracentrifuged serum.

Parameter	Moderate lipemia (TG: 700–1000 mg/dl)			Allowable bias (%)
	Native serum (%)^*∗*^	Serum blank (%)^*∗*^	Serum dilution (%)^*∗*^	
ALT	+10			**11.48**
Albumin	+7	0	+34	**1.43**
ALP	0			**6.72**
Amylase	+7			**7.4**
AST	0			**6.54**
Bilirubin (total)	0			**8.95**
Calcium	+32	+20	+34	**0.82**
Chloride	+35	+6	+33	**0.5**
CPK	−13	−11	−24	**11.5**
Creatinine	−7	0	−34	**3.96**
GGT	−30	−2	+144	**11.6**
Glucose	+16	0	+33	**2.34**
Iron	+11	+26	+41	**8.8**
LDH	+2			**4.3**
Lipase	+42	−20	+177	**11.31**
Magnesium	+146	+92	+123	**1.8**
Phosphorus	+106	+103	+128	**3.38**
Total protein	+26	−8	+32	**1.36**
TIBC	+13	+16	+34	**1.3**
Urea	−6	−6	−21	**5.57**
Uric acid	+2			**4.87**

^*∗*^Difference with ultracentrifuged serum, calculated as bias.

**Table 4:** Comparison of the results of native serum, serum blank, and diluted serum with ultracentrifuged serum.

Parameter	Severe lipemia (TG > 1000 mg/dl)			Allowable bias
	Native serum (%)^*∗*^	Serum blank (%)^*∗*^	Serum dilution (%)^*∗*^	
ALT	0			**11.48**
Albumin	+13	0	+43	**1.43**
ALP	−3			**6.72**
Amylase	+14	+4	+15	**7.4**
AST	+14	−27	+36	**6.54**
Bilirubin (total)	0			**8.95**
Calcium	+40	+30	+57	**0.82**
Chloride	+82	+22	+90	**0.5**
CPK	−22	−5	−61	**11.5**
Creatinine	−20	−80	0	**3.96**
GGT	−9			**11.6**
Glucose	+44	+2	+63	**2.34**
Iron	−9	+660	+21	**8.8**
LDH	+9	+8	+7	**4.3**
Lipase	+7			**11.31**
Magnesium	+180	+108	+325	**1.8**
Phosphorus	+109	+187	+210	**3.38**
Total protein	+59	−3	+70	**1.36**
TIBC	+34	+33	+78	**1.3**
Urea	+11	+17	−17	**5.57**
Uric acid	+4			**4.87**

^*∗*^Difference with ultracentrifuged serum, calculated as bias.

## Data Availability

The data used to support the findings of this study are included in the article. Previously reported data were used to support this study and are available at DOI: 10.11613/BM.2011.025 and DOI: 10.3343/alm.2018.38.6.518.

## Abbreviations

TG: Triglyceride
CLSI: Clinical and Laboratory Standards Institute
ALT: Alanine aminotransferase
ALP: Alkaline phosphatase
AST: Aspartate aminotransferase
CPK: Creatine phosphokinase
GGT: *γ*-Glutamyl transferase
LDH: Lactate dehydrogenase
TIBC: Total iron-binding capacity.
